# High School Students' Depression Literacy about Interventions and Prevention: A Survey in Tehran

**DOI:** 10.1155/2023/8540614

**Published:** 2023-03-03

**Authors:** Zahra Khorrami, Azadeh Sayarifard, Shahrbanoo Ghahari, Nadereh Memaryan, Mohammadreza Pirmoradi, Laleh Ghadirian

**Affiliations:** ^1^School of Behavioral Sciences and Mental Health, Iran University of Medical Sciences, Tehran, Iran; ^2^Knowledge Utilization Research Center, Center for Academic and Health Policy, Tehran University of Medical Sciences, Tehran, Iran; ^3^Department of Mental Health, School of Behavioral Sciences and Mental Health (Tehran Institute of Psychiatry), Iran University of Medical Sciences, Tehran, Iran; ^4^Spiritual Health Research Center, Mental Health Department, School of Behavioral Sciences and Mental Health (Tehran Institute of Psychiatry), Iran University of Medical Sciences, Tehran, Iran; ^5^Department of Clinical Psychology, Center of Excellence in Psychiatry, School of Behavioral Sciences and Mental Health, Iran University of Medical Sciences, Tehran, Iran; ^6^Community-Based Participatory Research Center, Iranian Institute for Reduction of High-Risk Behaviors, Center for Academic and Health Policy, Tehran University of Medical Sciences, Tehran, Iran

## Abstract

**Background:**

Given the high prevalence of depressive disorders in the present world and the lack of adequate awareness about prevention and appropriate interventions, increasing mental health literacy is vital for promoting mental health to reduce depression and its consequences.

**Methods:**

In this descriptive cross-sectional study, participants were recruited among the second high school students in the 2018-2019 academic year. The sample size was 2038, and samples were selected by multistage cluster sampling from different areas of Tehran. Demographic variables like age, gender, level of education, and parents' characteristics and mental health literacy questions in treatment and prevention areas were evaluated.

**Results:**

Analyses showed that of high school students, 83% considered getting help from psychiatrists and 80% considered learning stress management as the best preventive measures, while as the best treatment measures, 79.5% considered counseling the best place to refer for visiting a professional and 45% selected general counseling centers.

**Conclusion:**

The study results showed that high school students have a positive attitude toward preventing and treating depressive disorders, getting help from specialists, and useful measures for depressed people. But they did not know enough about preventive measures, including learning effective coping skills, reading self-help books, and continuing to take psychiatric medications. Planning and providing the necessary training are important, especially for high school students.

## 1. Introduction

Individuals are the most important asset of every society, and the health of a community is derived from the health of its human resources [1]. The World Health Organization (WHO) has identified four important dimensions of health definition, including physical, mental, social, and spiritual health [2]. One of the important aspects of health is mental health. By 2020, the burden of psychiatric disorders increased and accounted for 15% of disability-adjusted life years (DALYs). Depression was ranked fourth [3], while people do not refer to health care providers due to fear of judgment, high cost, lack of familiarity with people, and mental health providers [4]. According to the National Mental Health Survey in 2010-2011, the prevalence of psychiatric disorders in Iran was 23.6%, and of all patients with psychiatric disorders, only 20% received adequate treatment [5]. And studies show that the prevalence of serious psychological problems, including depression, among Iranian teenagers, varies between 14.77% and 72% [6]. A WHO study of 28 developed and developing countries showed that patients with depressive disorder had a delay with an average of 1–14 years for their first treatment [7]. Also, a study in Iran showed that 57.8% of patients referred to services only attended the first session and then discontinued treatment. Patients with a depressive disorder had the highest percentage of this discontinuation among the psychiatric disorders [8]. Although there are some effective and low-cost interventions for preventing and treating depression, the depression treatment gap is between 72% and 93% of all patients in low-income countries. Depression is a risk factor for addiction and chronic diseases like hypertension (HTN) and coronary heart disease (CHD). So, if treatment is not appropriate, in time, its health outcomes could worsen [9, 10]. Given this, the importance of timely preventive measures and proper therapies to prevent long-term complications is doubled. Countries should strive to promote health literacy in the communities to achieve this goal, especially mental health literacy. In defining mental health literacy, it can be said: “Mental health literacy is the individuals' knowledge and belief about mental disorders that can help them to identify, manage and prevent these problems” [11].

Generally, people are unaware of psychiatric disorders due to not being familiar with the symptoms and appropriate measures to prevent and treat them, so they do not recognize psychiatric disorders and seek treatment [12, 13]. In the field of prevention, people are more familiar with preventing physical illnesses such as diabetes and cardiovascular disease, when they are less familiar with the methods of preventing psychiatric disorders [14]. So, considering the concept of mental health literacy, improving depression literacy would lead to better identification, management, and prevention of this disorder. This study is aimed at evaluating the high school students' depression literacy by assessing their attitudes, beliefs, and general awareness about preventive measures and appropriate therapeutic interventions for major depressive disorder.

## 2. Materials and Methods

This research is a descriptive cross-sectional study. The results reported in this study are part of a study on the mental health literacy of secondary high school students in Tehran. The statistical population of this study was the second high school students of Tehran who were studying in the school year of 2018-2019. The sample size was 2038 people selected by the multistage cluster sampling method. To reduce selection bias for sampling, among the 19 education districts of Tehran, five districts were randomly selected from each specific area (north, south, east, west, and center of Tehran) to determine the effect of different disturbing variables that affect the research result. These areas were controlled, which were considered branches 2, 4, 7, 9, and 15, respectively, but because these clusters also included a large community, two schools (girls and boys) were randomly selected from each area. And from each of these schools, nine classrooms were selected; that is, we chose one class from each of the girls' and boys' schools, from each tenth, eleventh, and twelfth grade, and from each field of mathematics, experimental, and humanities. A total of 90 classrooms were selected. In each high school classroom, an average of 30 students were studying, and all students in these classes completed the questionnaires. Sample size (*n* = 384) was calculated based on the alpha error of 0.05 and power of 80% in the Morgan table considering three cluster coefficients [15]. A total of 2038 high school students participated in this study, taking into account the drop.

### 2.1. Measures

The variables assessed in this study were age, gender, participants' level of education and major, mother's and father's education years, and household income.

Depression literacy was evaluated using a scenario-based questionnaire that includes several areas, including diagnosis, prevention, treatment, first aid, specialists, related services, and stigma related to depression, anxiety, posttraumatic stress disorder, and schizophrenia. The questionnaire was the Australian Mental Health Literacy Questionnaire [16], and its validity and reliability in Iran were calculated by Sayarifard et al. [11].

The high school students in person completed the questionnaire. Because of the high burden of depressive disorder in society and the importance of preventive measures and early treatment, this study dealt with depression literacy in the prevention and treatment fields. At first, the depressive disorder scenario was given to the individual, and then, the related questions were asked. According to the country's culture, she or he characters were considered in the scenario for female or male high school students. The purpose of the study was explained to the high school students in advance, and they were completely free to enter or leave the study at any stage.

The scenario of the questionnaire (major depressive disorder vignette):

“She/He is 17 years old. She has been feeling sad and helpless for several weeks. She/He is always tired almost every night. She/He has trouble sleeping. He has lost weight and cannot concentrate on studying. He cannot decide, and even daily affairs seem beyond his ability. Her/His parents and friends are very worried about her/him.”

### 2.2. Statistical Analysis

Researchers hypothesized the relationship between demographic variables and depression literacy in treatment and prevention areas.

To determine the overall score of the two areas of prevention and intervention, agree response was recoded to 1, and disagree and neutral were recoded to 0. Hence, according to the number of items, the total score was between 0 and 13.

Then, ANOVA and *t*-test were used to analyze the relationship between the mental health literacy score in the prevention and intervention areas with qualitative variables. At the same time, the Pearson correlation was used to analyze the relationship between the total score and quantitative variables. A *P* value less than 0.05 was considered statistically significant.

This research has the ethics code of the Iran University of Medical Sciences and Health Services (IR.IUMS.REC.1396.9411704005).

## 3. Results

Finally, 2284 high school students filled out the questionnaire; 246 questionnaires were incomplete and removed, so the results of the 2038 questionnaires were analyzed. The mean age of participants was 16.7 ± 1.2 years, 1008 (49.5%) were girls, and 1030 (50.5%) were boys. The average years of education received by the participants' mothers and fathers were 11.6 ± 3.8 and 12 ± 4, respectively. The average household income was $502.7 ± 162.4 per month.

The number of participants studying mathematics was 633 (31.1%), experimental science was 716 (35.1%), and humanities was 689 (33.8%). These high school students were studying in the second half of high school, and of them, 657 (32.2%) high school students were in the tenth year, 695 (34.1%) high school students in the eleventh year, and 686 (33.7%) high school students in the twelfth year.

Participants' views on who is better to ask for help when they have a major depressive disorder are listed in Table 1.

Answers to questions about participants' beliefs and knowledge about the items that would reduce the risk of developing a problem, like a vignette, are in Table 2.

Participants' beliefs and knowledge about interventions that are likely to be helpful for the vignette are in Table 3.

In response to the question about the preference of referring to different types of counseling centers, general counseling centers 909 (45%), student counseling centers 510 (25%), private clinics 249 (12%), and telephone consultations 164 (8%) were selected.

The analytical results of the research hypothesis about assessing the existence of a relationship between gender, age, the field of study, educational level, socioeconomic status of the family, and education level of parents with mental health literacy about therapeutic interventions or prevention for the person with depressive disorder are in Tables 4 and 5.

Analyses showed that there was a significant relationship between the depression health literacy score in prevention and interventions with gender (*P* = 0.004), the field of study (*P* < 0.001), educational level (*P* = 0.013), and age (*P* = 0.035).

ANOVA analysis of mental health literacy score based on the field of the study showed an *F* equal to 11.26. Tukey's post hoc analysis showed that the score of experimental science high school students compared to mathematics and humanities high school students was significantly higher (*P* = 0.019 and *P* < 0.001).

Analysis of the mental health literacy score based on education years by ANOVA showed *F* equal to 4.38. In Tukey's post hoc analysis, the score of high school students in grade 12 compared to high school students in 10^th^ grade was significantly higher (*P* = 0.01).

The correlation between high school students' parents' education level and household income with mental health literacy about therapeutic interventions or prevention for depressive disorder did not show any significant differences.

## 4. Discussion

Depressive disorders are characterized by sadness, loss of interest or pleasure, guilt or low self-worth, disturbed sleep or appetite, tiredness, and poor concentration. Depression can be long-lasting or recurrent, substantially impairing an individual's ability to function at work or school or cope with daily life. At its most severe, depression can lead to suicide [17]. In many countries, the highest rate of suicide occurs among young people; globally, suicide is the second leading cause of death in the age group of 15 to 29 years [18]. Depression is one of the most important risk factors for suicide.

Furthermore, mental health problems in children and adolescents impose a heavy disease burden on societies, necessitating preventive and early treatment interventions [19]. One of the causes of disability globally is mental disorders [20]. Recognizing depression is one of the factors that helps people in the timely diagnosis of symptoms to receive specialized services and helps to reduce the risk of suicide and long-term suffering from this disorder and prevent its consequences [21]. Communities can help people identify and receive appropriate measures and services by promoting mental health literacy. According to the mentioned points, it is essential to help adolescents receive psychological services timely when they need such services; hence, it is helpful to be aware of adolescents' beliefs in this field to assess the need for some interventions, such as related training.

But to do essential planning in this regard, the first step is to determine the community's literacy level and awareness about psychiatric disorders like major depression. The purpose of the present study was to evaluate high school students' mental health literacy about mental health interventions and the effective prevention of major depressive disorder.

Because high school students are considered one of the most important and sensitive groups in each community, promoting mental health literacy is also essential for community health economics. One of the questions from high school students was about various mental health professionals who can help a person with depression. In response to who was the best person to help people with depression, most participants agreed with the help of a psychologist, a psychiatrist, and then a family physician. In Sayarifard et al.'s study on medical high school students in Tehran, most students chose psychiatrists, psychologists, and family physicians [11]. This difference may be due to the community stigma about psychiatric medication use. Also, high school students may be more interested in referring to psychological services because of lower awareness about medication than medical high school students. In both studies, getting help from a family physician and general practitioner was the subjects' last priority; this could be attributed to the importance of specialization and more tendency to refer to specialists rather than general practitioners [22] and the shortcomings in implementing the family physician program [23].

In Jorm et al.'s study, 87.3% of Australian youth chose to refer to a family physician as the best option [24], which could be due to the successful and high quality of family physician services in this country [25]. In Beaudry et al.'s study of adolescents in America, the choice between a psychologist and a psychiatrist was almost equal [26]. The reason could be the similar quality of service delivery by both groups and equal stigma about them.

In the present study, 71.3% of the respondents prioritized getting help from a family member. Still, given the importance of the peer group in adolescence, 72.9% preferred to seek help from their close friends. In Ghadirian and Sayarifard's study on Tehran residents [27], 41% agreed to receive support from family members, and 46.5% preferred a close friend, while residents of Tehran's suburbs were more likely to seek help from a close friend. Findings were aligned with this study, so improving peer group mental health literacy through formal education programs could be helpful. Since having close family relationships is the dominant culture in Iranian society, children find the family environment a safe place; therefore, they seek help from family members in case of problems.

In this study, 70.1% agreed with getting help from a school counselor. In Beaudry et al.'s investigation, around 50% [26], and in Jorm et al.'s study [24] comparing Australian and Japanese youth, 82.2% of Australian youth, and 85.8% of Japanese youth agreed with the help of the counselors. Differences may depend on the emphasis and importance of the counselor position at the schools and the quality of delivered services to address the high school students' problems in each country.

Given that the majority of Iranian adolescents agree to receive school counseling, and school counseling is the most accessible service for this group, it is appropriate that school counselors focus on promoting mental health literacy rather than being educational counselors since, in Iran, because of the importance of the university entrance exam, much of the school counseling activities are geared toward this issue.

Another question from high school students was about prevention and items that prevent the progression of depression. Most subjects agreed that they should learn how to avoid stressful situations and manage that if they get stressed. The following best options were close relationships with family members and, after that, close friends. These results were similar to Sayarifard et al.'s study [11] on medical high school students. Finally, 57.3 percent agreed with learning effective coping skills, which was lower than other prevention options. Considering that effective coping skills in dealing with stressful situations are among the most effective ways of preventing psychiatric disorders, and noting that perhaps high school students' lack of understanding these mechanisms is the reason for this finding, implementing appropriate educational interventions focused on coping skills is essential.

In response to the question about proper measures for people with depression, in the present study, most of the high school students chose counseling as the best approach, which was similar to the results of medical high school students in Tehran, which reflected a positive attitude of high school students in this regard. Therefore policymakers and planners need to consider providing accessible and high-quality counseling services for high school students. The majority of adolescents agreed that physical activity is a beneficial action for patients with depression, which was comparable with Parker et al.'s [28], Sayarifard et al.'s [11], Yoshioka et al.'s [29], and Loureiro et al.'s [30] study. In the present study, 71.7% chose to perform relaxation techniques as a useful measure that was consistent with Sayarifard et al.'s [11]; Parker et al.'s, both Australian and Singapore youths [28]; and Loureiro et al.'s [30] study. The lowest selected approach for depressed patients was reading self-help books, among valuable measures.

One of the most critical components for the survival of societies is the component of culture, and to promote a society, it must improve. On the other hand, reading is one of the culture-promoting factors in the community. Reading skill level is low in Iranian society for various reasons, such as unfamiliarity and not being accustomed to reading in childhood and adolescence [31].

The studies on children and adolescents' interest in reading printed books showed that teens found it boring to read printed books and believed using them belonged to the past [32]. Nowadays, considering scientific and technological advances, replacing old with modern media such as e-books and audiobooks can help raise people's awareness [33].

In addition, the high cost of buying books in Iran is another factor that plays a role in low public motivation to study printed books, and subjects tend to look at websites instead of printed books.

With the advancement of technology, it has become commonplace to think that access to the Internet can provide much information in different areas. Still, it is worth pointing out that all contents on the various websites are not sufficiently monitored and may be incorrect [34].

A small percentage of high school students chose to join support groups as a practical measure, which could be due to the lack of support groups for psychiatric patients in the community, resulting in no familiarity with these groups.

The study on the impact of online support groups on depressive disorder has revealed that the benefits and advantages of participation in these groups were communication with others, depression normalization, and labeling reduction. However, the level of involvement and membership in online support groups is generally low [35]. Obstacles such as the fear of being judged, the feeling of being different from the group members, and the fear of hurting others or themselves are always present. But these groups could further reduce the consequences of depression and stigma [35].

Another question was, “After five days of prescribed medication, should the patient continue to take the medication if the patient does not feel tangible improvement?” Only 35.3% of participants agreed to continue taking the drug, which means that about one-third of the participants had a correct attitude toward psychiatric medication use. It shows that educating people about the proper use of psychiatric medication is essential.

In an answer to the question “which health centers do you prefer to get mental health services?”, high school students believed that general counseling and student counseling centers were the best and most accessible centers for these services which were the same as the results of Sayarifard et al.'s study on medical high school students [11]. Only 12 percent considered private clinics as the best choice; that could be due to the high cost of private psychological services and perhaps the lack of easy access to these centers, and given that the adolescent may not have enough freedom to make this choice, so they should be referred to these centers under parental supervision. Only 8% of the participants chose telephone counseling despite its easy access. Given that the use of telephone consulting services is available and fast, it is less accepted; that could be due to the lack of interaction between the client and the therapist, which greatly impacts the quality of counseling. Most people tend to consult in person, but because of the lower cost and availability of virtual consulting using advanced technology, telephone counseling can be replaced with online consulting, such as video and web-based services, to address this problem.

Men are less likely to seek health information and services than women because of their more pessimistic view of psychiatric disorders [36]. The present study results also showed a relationship between the depression literacy score in prevention and interventions with gender. Perhaps this is due to epidemiologic findings that showed a higher prevalence of depression among women than men [37]. Concerning the importance of women's health in society, communities have encouraged this group to raise their awareness of depression disorder. As a result, it has promoted women's knowledge about prevention and familiarity with appropriate interventions and services.

There was also a significant relationship between the field of study and mental health literacy scores regarding preventive and therapeutic interventions for depressed individuals. High school students studying experimental sciences had higher health literacy than humanities and mathematics sciences, which could be because health-related issues are the main educational concepts in this field. There was also a significant relationship between depression literacy in interventions and prevention with school year and age, which was consistent. Based on the studies that showed a meaningful relationship between overall health literacy and the school year, the higher the academic year, the higher the health literacy [38, 39].

The present study investigated the correlation between high school students' age and depression health literacy regarding therapeutic and preventive interventions. No significant relationship was shown, contrary to Ghadirian and Sayarifard's study of depression health literacy in the general population aged 18-68. In this study, higher age and education years are predictors of better depression literacy [27]. We can explain this finding to the limited high school students' age range (15-19 years) in the present study. Being a teenager can also reflect the low experience of high school students with psychiatric disorders [40].

Harley's study showed a significant relationship between parents' education level and their children's mental health [41]. However, there was no significant relationship between parents' education level and high school students' depression health literacy in the present study, which is consistent with Stormacq et al.'s study that mentioned the parents' education degree and issues such as communication skills, the quality of communication between parents and high school students, and the hours spent with parents in this age group. It may not be entirely accurate to measure parents' education based solely on school years, meaning that people with the same level of education may have different cognitive levels [42]. So adolescents spend less time with their parents, and parental control and influence decrease with age. In addition, the effects of peers becoming more prominent [43] offer to teach the components of mental health literacy to them by themselves as a suggested solution. In the present study, there was no significant relationship between household income and high school students' depression health literacy, which may be due to the age of the participants and lack of knowledge about the exact family income [44].

## 5. Conclusion

This study revealed that participants in the prevention and treatment of depressive disorder had a positive attitude about getting help from specialists and the effectiveness of measures. Most participants selected psychologists, psychiatrists, family support, and friend groups as the best sources for this issue. They also found it more appropriate to visit public counseling centers if they had a depressive disorder. But their knowledge about preventive measures, including learning effective coping skills, reading self-help books, and continuing psychiatric medication use, was insufficient. So it is essential to provide the necessary training, especially concerning the student group. The results of this research can be helpful in future planning, what should be provided in the field of service quality, and the contents of training to address deficiencies in this area.

### 5.1. Strengths and Limitations

Samples were selected from different city areas, and various confounders' roles were reduced using randomization. Given the large sample size, study findings have high generalizability.

But because these findings are from adolescents' views and given their age, their decision to perform any measure to prevent or treat depression depends on their parent's decision. So these findings are not necessarily what they will do in the case of depression.

It is suggested that future studies in Iran be focused on identifying the causes of deficiencies found in the present study to find effective interventions to correct them.

## Figures and Tables

**Table 1:** Adolescent's beliefs about people who could possibly help the vignette.

People	Agree (number (%))	Disagree (number (%))	Neutral (number (%))
Psychologist	1693 (83.1)	105 (5.2)	238 (11.7)
Psychiatrist	1332 (65.5)	231 (11.3)	473 (23.2)
General practitioner or family doctor	1208 (59.3)	174 (8.5)	655 (32.2)
Close family member	1453 (71.4)	197 (9.7)	384 (18.9)
Close friend	1485 (72.9)	160 (7.8)	393 (19.3)
Teacher	878 (43.1)	312 (15.3)	846 (41.6)
School counselor	1425 (70.1)	155 (7.6)	454 (22.3)
Clergyman	367 (18)	920 (45.2)	749 (36.8)
Pharmacist	126 (6.2)	1067 (52.4)	842 (41.4)
Seller of herbal medicine	299 (14.7)	865 (42.6)	868 (42.7)

**Table 2:** Adolescent's beliefs and knowledge about the items would reduce the risk of developing a problem like a vignette.

Item	Agree (number (%))	Disagree (number (%))	Neutral (number (%))
Learning to avoid becoming stressed in the first place and taking actions to decrease stress when it occurs	1622 (79.7)	205 (10.1)	209 (10.3)
Keeping regular contact with friends	1209 (59.4)	309 (15.2)	518 (25.4)
Keeping regular contact with family	1417 (69.6)	314 (15.4)	304 (14.9)
Learning coping skills	1167 (57.3)	305 (15)	563 (27.7)

**Table 3:** Adolescent's beliefs and knowledge about interventions that are likely to be helpful for vignette.

Intervention	Agree (number (%))	Disagree (number (%))	Neutral (number (%))
Becoming more physically active	1590 (78.2)	54 (2.7)	390 (19.2)
Getting relaxation training	1459 (71.7)	64 (3.1)	513 (25.2)
Receiving counseling	1617 (79.5)	101 (5)	317 (15.6)
Reading a self-help book on (his/her) problem	1355 (16.6)	158 (7.8)	522 (25.7)
Joining a support group of people with similar problems	694 (34.1)	423 (20.8)	921 (45.2)
Looking up a website giving information about (his/her) problem	902 (44.3)	425 (20.9)	711 (34.9)
Going to a local mental health service	895 (44)	339 (16.7)	801 (39.4)
Being admitted to a psychiatric ward of a hospital if necessary	605 (29.7)	953 (46.8)	477 (23.4)
Continuing treatment if the vignette does not feel any tangible improvement after five days of using the prescribed medication	717 (35.3)	643 (31.6)	674 (33.1)

**Table 4:** The relationship between depression health literacy score with gender, the field of study, and educational level.

Variable	Mean ± SD	*P* value
Gender		
Female	7.7 ± 2.4	0.004
Male	7.3 ± 2.5	
Field of study		
Experimental science	7.8 ± 2.4	<0.001
Mathematics	7.4 ± 2.5	
Humanities	7.2 ± 2.5	
Educational level (school grade)		
10	7.3 ± 2.5	0.013
11	7.6 ± 2.5	
12	7.7 ± 2.4	

**Table 5:** Correlation between high school students' age, their parent's education level, and household income with mental health literacy.

Variable	Pearson correlation coefficient	*P* value
Age	0.05	0.035
Mother's education years	0.03	0.15
Father's education years	0.02	0.47
Household income	-0.01	0.6

## Data Availability

The data used to support the findings of this study are available from the corresponding author upon request.
